# Impact of Flavonoid-Enriched Antioxidant Nanoformulation Supplementation on In Vitro Maturation and Gene Expression of Buffalo Oocytes

**DOI:** 10.3390/ani15081147

**Published:** 2025-04-16

**Authors:** Eman M. El-Saka, Abou Bakr A. El-Wishy, Adel R. Moawad, Sally Ibrahim, Saber Ibrahim, Abdallah M. Shahat

**Affiliations:** 1 Department of Theriogenology, Faculty of Veterinary Medicine, Cairo University, Giza 12211, Egypt; eman.elsaka@vet.cu.edu.eg (E.M.E.-S.); abdallah_shahat@cu.edu.eg (A.M.S.); 2 Division of Animal Science, College of Agriculture, Family Sciences, and Technology, Fort Valley State University, Fort Valley, GA 31030, USA; 3 Department of Animal Reproduction and AI, Veterinary Research Institute, National Research Centre, Giza 12622, Egypt; sally_rashad2004@yahoo.com; 4 Packaging Materials Department, National Research Centre, Dokki, Giza 12622, Egypt; msa.nrc@gmail.com; 5 Nanomaterials Investigation Lab., Central Laboratory Network, National Research Centre, Dokki, Giza 12622, Egypt

**Keywords:** buffalo, oocyte, IVM, antioxidants, flavonoids, gene expression, nanoparticles

## Abstract

Oocytes are exposed to various stressors during in vitro maturation (IVM). Antioxidant supplementation during IVM can mitigate oxidative stress. We investigated the effects of supplementing IVM medium with novel flavonoid-enriched antioxidant nanoformulations, namely, EMD-300^®^ and EMP3-H200^®^, on oocyte IVM and analyzed the expression of oxidative stress, apoptosis, and pluripotency genes in buffalo. Cumulus oocyte complexes (COCs) obtained from buffalo ovaries were matured in IVM medium supplemented with either EMD-300^®^ or EMP3-H200^®^ at 0.5% and 1.0% for 22 h. Following IVM, nuclear maturation, gene expression, and the levels of total antioxidant capacity (TAC) and malondialdehyde (MDA) were analyzed. Nuclear maturation was lower (*p* < 0.001) for the 1.0% EMD-300^®^ group than other groups. The expressions of the GPX4, SOD, CAT, and ATF6 genes were lower (*p* < 0.05) in the 0.5% EMD-300^®^ and EMP3-H200^®^ groups than in the control. OCT4 gene expression was higher (*p* < 0.05) for the treated groups than control group. The level of TAC in spent IVM medium was higher for the 0.5% EMD-300^®^ and EMP3-H200^®^ groups than for the control. However, the MDA concentrations were lower. In conclusion, supplementing IVM medium with EMD-300^®^ or EMP3-H200^®^ at 0.5% improved nuclear maturation of buffalo oocytes better than 1.0%. Our findings suggest that these compounds had antioxidant effects, which assures their ability in protecting oocytes against oxidative stress.

## Simple Summary

Oocytes are exposed to different stressors during in vitro culture, which negatively impacts the developmental competence. Antioxidant supplementation during in vitro maturation (IVM) counteracts the detrimental effects of oxidative stress. We studied the effects of novel flavonoid-enriched antioxidant nanoformulations, namely, EMD-300^®^ and EMP3-H200^®^, on oocyte IVM and the expression levels of some genes associated with antioxidant activity, apoptosis, and pluripotency in buffalo. We found that supplementation of the IVM medium with either 0.5% EMD-300^®^ or 0.5% EMP3-H200^®^ is associated with a reduction in oxidative stress and improvement in oocyte quality, as confirmed by changes in the gene expression patterns.

## 1. Introduction

Domestic water buffaloes (*Bubalus bubalis*) are a worldwide significant livestock species that are well adapted to tropical and subtropical climates, ensuring their place in future food security, especially with the growing human population and climate changes. Furthermore, they are more capable of converting low-quality roughages, agriculture byproducts, and residues into milk and meat [1,2]. Compared to cattle, buffaloes have low reproductive performance, mainly due to weak estrus expression, fewer numbers of primordial follicles, and a higher incidence of follicular atresia [3,4]. These physiological constraints constitute a major impediment to the full exploitation of buffaloes’ productive capacity. In vitro embryo production (IVEP) is a valuable reproductive technology that could potentially help in the genetic improvement and maximize the production of water buffalo [5]. Despite the advancement of IVEP in buffaloes, with in vitro maturation (IVM), cleavage, and blastocyst rates of 95.8%, 75.2%, and 33.4%, respectively, the development of oocytes to the blastocyst stage remains insufficient compared to cattle [5]. This limitation is primarily due to the inferior quality of in vitro matured oocytes compared to those matured in vivo [6]. As a result, it is critical to focus on the maturation process and explain how it affects the quality and efficiency of oocytes, with the aim of improving their capacity to complete subsequent steps to achieve a high blastocyst rate.

It is well known that the bidirectional communication between the oocytes and neighboring cumulus cells plays a crucial role in oocyte growth, meiotic maturation, and fertilization [7]. Cumulus cells connect to the cytoplasm of the oocyte and pierce the zona pellucida through the gap junctions allowing for bidirectional paracrine signaling and transfer of tiny particles that are essential for oocyte maturation [8]. Furthermore, cumulus cells assist the oocyte in its metabolic processes and its capacity to control oxidative stress [9].

Oocyte IVM is the first step of IVEP and the starting process for many applications of assisted reproductive technologies (ARTs), including in vitro fertilization (IVF), somatic cell nuclear transfer (SCNT), transgenesis, and stem cell research [10]. Oocyte maturation is a complex process through which oocytes gain the ability to support embryo development and activate embryonic DNA. It involves two phases: nuclear and cytoplasmic maturation [11]. Nuclear maturation mainly comprises chromosomal segregation, and cytoplasmic maturation includes organelle reorganization and storage of mRNA, proteins, and the transcription factors required for fertilization and subsequent early embryonic development [12].

Handling of gametes and embryos in vitro generates high levels of reactive oxygen species (ROS) that surpass the physiological antioxidant capacity of the cell, resulting in the induction of oxidative stress [13]. It is well known that buffalos’ oocytes are highly vulnerable to oxidative stress during IVM, mainly due to their high lipid content [5]. Various factors, including high oxygen concentrations during incubation, exposure to light, and environmental pollutants, have been reported to generate high ROS levels and subsequent oxidative stress in oocytes during IVM [14,15,16]. This can consequently cause perturbations in spindle and chromosomal configuration, apoptosis, and DNA fragmentation, which negatively impact the developmental potential of oocytes following IVM, IVF, and in vitro culture (IVC) [13].

Antioxidants are substances that help to minimize the bimolecular oxidation or prevent it from occurring in the early stages [17]. They consist of a variety of both natural and synthetic substances and can counteract ROS and decrease the severity of free radical oxidation, thus safeguarding cell membranes and DNA within the cells and mitochondria [18]. Antioxidant supplementation during IVM has been proposed as a strategy to overcome the detrimental effects of oxidative stress, maintain ROS at physiological levels, and improve oocyte quality [19]. Various antioxidants include thiol compounds like cysteamine and β-mercaptoethanol have been utilized during IVM of buffalo oocytes to improve their quality by boosting glutathione levels [20,21]. Additionally, natural compounds have also shown promising results on oocyte quality and IVM. Natural antioxidants, especially those rich in essential oils, can be utilized as an alternative to minimize the negative effects of oxidative stress in culture media [22]. These oils possess a range of bioactive compounds with antioxidant, antimicrobial, and anti-inflammatory activities [23].

Flavonoids are a group of natural substances that possess different biological functions among them the antioxidant activity [24]. The antioxidant capacity of flavonoids is attributed to their ability to scavenge ROS and the subsequent enhancing the synthesis of intrinsic antioxidant enzymes such as superoxide dismutase (SOD) and glutathione (GSH) [25]. Several flavonoids were used in vitro to improve the quality and competence of oocytes in different species, including buffalo [26], cattle [27,28], and sheep [29]. In buffalo, it has been reported that supplementing IVM media with 10 µg/mL kaempferol, a potent flavonoid-enriched antioxidant, improved oocyte maturation rates compared to the control (87.08% vs. 66.32%, respectively) [26]. In cattle, previous studies showed that adding quercetin (2 µM) or resveratrol (2 µM) to the IVM medium significantly reduced the levels of ROS in matured oocytes [27]. Moreover, it has been demonstrated that supplementing IVM medium with nobiletin, a polymethoxylated flavonoid, at 25 μM or 50 μM, enhanced bovine oocyte nuclear maturation, cleavage, and blastocyst rates following IVM/IVF and embryo culture compared to the control (without nobiletin supplements) [28]. In the same study the authors found that nobiletin supplementation also improved mitochondrial activity and reduced the levels of ROS and GSH in matured oocytes [28]. In sheep, it has been demonstrated that inclusion of grape seed extract (800 µg/mL) or quercetin (5 or 15 µg/mL) during IVM imposed beneficial effects on oocyte maturation and subsequent developmental potential following IVF and embryo culture [29]. These studies assure the essential role played by flavonoid-enriched natural antioxidants on the quality, maturation, and in vitro development of oocytes in different species, as well as their roles in protecting the oocytes against oxidative stress and ROS production. Therefore, in the present study two naturally flavonoid-enriched antioxidant compounds extracted from Egyptian native plants were tested on buffalo oocyte IVM.

In recent years, nanotechnology has been efficiently utilized in ARTs to improve oocyte maturation, fertilization, and in vitro embryo development [30,31,32]. Nanoparticles (NPs) possess unique physical characteristics that are different from those of microparticles and bulk materials [31]. These characteristics include smaller size, larger surface area, higher purity, increased stability, and mutual interactions at fluid interfaces, allowing NPs to be incorporated as potential candidates for enhancing IVEP [31,32]. For instance, supplementation of IVM medium with selenium nanoparticles (SeNP) improved buffalo oocyte nuclear maturation through up regulation of antioxidant defense and pluripotency gene expression [33,34]. Moreover, treatment of cattle oocytes with melatonin-loaded lipid-core nanocapsules (Mel-LNC) during IVM improved the quality of in vitro produced blastocytes [35]. In addition, in cattle, a previous study showed that inclusion of nano-selenium (NSe) and nano-zinc oxide (NZn-O) during IVM significantly increased both intracellular GSH concentration and the DNA integrity of the cumulus cells [36]. The above-mentioned studies highlight the critical role played by NPs as enhancers for in vitro oocyte maturation and subsequent in vitro embryo development.

The aim of this study was to investigate the effects of supplementing IVM media with novel flavonoid-enriched antioxidant nanoformulations on oocyte quality, in vitro maturation rates, and the expression levels of some genes associated with oxidative stress, apoptosis, and pluripotency in buffalo.

## 2. Materials and Methods

Unless stated otherwise, all chemicals and reagents were purchased from Sigma Aldrich (St. Louis, MO, USA). All experimental procedures were approved by the Animal Ethical Committee, Faculty of Veterinary Medicine, Cairo University (Giza, Egypt) (Vet CU 18042024925). A two naturally flavonoid-enriched antioxidant compounds extracted from Egyptian native plants were used in the current study. The compounds were named EMD-300^®^ and EMP3-H200^®^. The formula of these compounds is still under disclosure as they were submitted for a product patent (EG/P/2025/20). They are biocompatible, biodegradable, safe, and eco-friendly antioxidant and antibacterial nanoformulations. These compounds were supplied by the Nanomaterials Investigation Laboratory, Central Laboratory Network, National Research Centre, Egypt (Giza, Egypt).

### 2.1. Characterization and Particle Size of EMD-300^®^ and EMP3-H200^®^

The particle size and zeta potential of EMD-300^®^ and EMP3-H200^®^ were analyzed using a NICOMP 380 ZLS (PSS, Santa Barbara, CA, USA). The particle size was measured according to dynamic light scattering with 700 measurements of the collision laser beam with the EMD-300^®^ and EMP3-H200^®^ particles. Zeta potential was measured as particle charge through an applied electrical current on alloy electrodes. The average particle size was 490 nm for EMD-300^®^ and 173 nm for EMP3-H200^®^.

### 2.2. 2,2-Diphenyl-1-Picrylhydrazyl Radical (DPPH) Scavenging Assay

2,2-Diphenyl-1-Picrylhydrazyl (DPPH) is a stable free radical used mainly as a sensitive colorimetric free radical scavenger and antioxidant detector. It is utilized to measure the radical scavenging activity of antioxidants by reducing its odd electron to form hydrazine [37]. A DPPH scavenging assay is widely used to determine the antioxidant activity of crude extracts or essential oils extracted from plants. The antioxidant activity of EMD-300^®^ and EMP3-H200^®^ were measured by mixing 10, 20, or 30 μg/mL with a methanol solution and 3 mL of DPPH radicals. The mixture was shaken vigorously and then left in the dark for 30 min. The absorbance of the combination was determined at 517 nm with a Shimadzu UV-160-IPC spectrophotometer against a blank. The following formula was used to calculate the results: I% = [(ΔA517C − ΔA517S)/ΔA517S]. ΔA represents the average absorbance, C the control, and S the sample [37]. Among the different concentrations used, 30 μg/mL produced the most potent effect for reduction of DPPH for both EMD-300^®^ and EMP3-H200^®^ with an efficiency of 79.89% and 89.37%, respectively.

### 2.3. Collection of the Ovaries and Cumulus–Oocyte Complexes (COCs)

Ovaries were obtained from apparently healthy buffalo female reproductive organs within 30 min of slaughter from EL-Sharkawy abattoir, Qalubia, Egypt from March to October 2024. The ovaries were kept in a thermos flask containing pre-warmed 0.9% isotonic saline at 25–30 °C and transported to the laboratory within 2 h. Ovaries were then washed in a warm saline to remove blood and debris, then they were kept in a beaker and maintained at 37 °C until oocyte retrieval [38]. Cumulus–oocyte complexes (COCs) were aspirated from 4–8 mm follicles using an 18-gauge needle attached to a 10-mL syringe. The follicular fluid containing the COCs was transferred to 15-mL conical tubes and incubated at 37 °C for 15 min to allow the COCs to settle at the bottom. The upper layer of the follicular fluid was then removed, and 5 mL of the fluid containing the COCs was transferred to a 90-mm Petri dish containing an oocyte washing medium, composed of HEPES-TCM199 (Thermo Fisher Scientific, Wilmington, DE, USA) supplemented with 10% *v*/*v* fetal bovine serum (FBS). Oocytes were examined under a stereomicroscope (AmScope, Irvine, CA, USA) and classified into good quality and bad quality oocytes. The good quality oocytes possess at least two compact layers of cumulus cells, and a homogeneous dark ooplasm. Meanwhile, the bad quality oocytes have less than two layers of cumulus cells, fluffy or expanded cumulus cells, or denuded with pale or heterogeneous granulated ooplasm [39]. The good quality oocytes were selected for in vitro culture (culturable), while the bad quality oocytes were discarded and not selected for IVM (non-culturable).

### 2.4. Oocyte IVM

COCs were washed three times in IVM medium composed of TCM199 (Thermo Fisher Scientific, Wilmington, DE, USA) supplemented with 10% FBS, 5 μg/mL FSH, 10 μg/mL LH, and 50 µg/mL gentamycin sulphate. Twenty to twenty-five oocytes were cultured in vitro for 22 h in 100 μL drops of IVM medium covered with sterile mineral oil (Avi Chem Laboratories, Bhiwandi, India) that had been equilibrated for 2 h at 38.5 °C in a humidified atmosphere with 5% CO_2_. The IVM medium was supplemented with either EMD-300^®^ or EMP3-H200^®^ at concentrations of 0.5% or 1.0%. COCs that were cultured in IVM media without EMD-300^®^ or EMP3-H200^®^ supplementation were considered as the control. Based on the results of cumulus expansion and nuclear maturation rate, the best concentration from EMD-300^®^ and EMP3-H200^®^ treatments was selected for conducting gene expression analysis and measurement of total antioxidant capacity (TAC) and malondialdehyde (MDA) levels.

### 2.5. Evaluation of Cumulus Expansion and Nuclear Maturation

Following IVM, the percentage of oocytes with expanded cumulus cells (widely expanded cumulus with loads of elastic intercellular matrix and loosened cumulus cell layers) was assessed under a stereomicroscope [40]. For evaluation of nuclear maturation, cumulus cells were removed by repeated pipetting. Then the oocytes were examined under an inverted microscope at 20× magnification for the identification of the 1st polar body (1st Pb) in the perivitelline space [41]. Denuded oocytes, as well as cumulus cells left from the COCs, matured in medium containing the optimal concentration of EMD-300^®^ or EMP3-H200^®^ were washed once in 30–40 µL 1× PBS. Denuded oocytes were transferred to a different drop of PBS. Oocytes and their respective cumulus cells were then collected separately in 1.5 mL Eppendorf tubes. The tubes were snap frozen in liquid nitrogen vapor and then kept at −80 °C for gene expression analysis. Furthermore, the spent maturation medium from each experimental group was collected following IVM and stored at −20 °C for measuring the levels of TAC and MDA.

### 2.6. RNA Isolation and cDNA Synthesis

Total RNA was extracted from the matured oocytes and their cumulus cells from the control and EMD-300^®^ or EMP3-H200^®^ treated groups (15–18 pooled oocytes per replicate, 4 independent replicates, total number of oocytes/group = 71) using the miRNeasy Mini Kit (Qiagen, Hilden, Germany), following the manufacturer’s protocol. Any potential contamination of genomic DNA was removed through on column DNA digestion using the RNase free DNase Kit (Qiagen, Hilden, Germany). The extracted total RNA was then stored at −80 °C. The concentration of total RNA was measured using a NanoDrop 2000/c (Thermo Fisher Scientific, Wilmington, NC, USA), and the integrity of the RNA was verified through 2% agarose gel electrophoresis. Samples displaying clear 28S and 18S ribosomal RNA bands were selected for gene quantification. cDNA was synthesized from the isolated total RNA using the RevertAid First Strand cDNA Synthesis Kit (Thermo Fisher Scientific, Wilmington, NC, USA). The RNA concentration was adjusted with RNase free water to a final volume of 10 µL. Then, 1 µL of oligo (dT) 18 and 1 µL of random primers were added, bringing the total volume to 12 µL, and the mixture was kept on ice briefly. Subsequently, a reaction mixture containing 4 µL of 5× reaction buffer, 1 µL of Ribolock RNase inhibitor, 2 µL of dNTP mix, and 1 µL of RevertAid RT was prepared. Eight microliters of this mixture were added to each of the RNA samples and mixed thoroughly by pipetting. A thermocycler was programmed for the following conditions: 25 °C for 5 min, 42 °C for 60 min, 70 °C for 5 min, and a final hold at 4 °C. The synthesized cDNA was then verified by PCR using a GAPDH primer and stored at −20 °C.

### 2.7. Quantitative Real Time PCR (qRT PCR)

Gene-specific primers for catalase (CAT), glutathione peroxidase 4 (GPX4), superoxide dismutase (SOD), activating transcription factor 6 (ATF6), caspase 3 (CASP3)*,* octamer-binding transcription factor 4 (OCT4), and glyceraldehyde-3-phosphate dehydrogenase (GAPDH) were designed using the Primer3 Program version 4.0 (Table 1) [42]. The real time PCR was conducted in a 20 μL reaction volume, containing Maxima SYBR Green/ROX qPCR Master Mix (2×) (Thermo Fisher Scientific, Wilmington, NC, USA) and 2 μL cDNA template. Real time PCR was performed using a Stratagene Mx3000P™ Real-Time PCR system with the following thermal cycling conditions: 95 °C for 10 min, followed by 45 cycles of 95 °C for 15 s, 60 °C for 30 s, and 72 °C for 30 s. This was followed by a dissociation (melting) curve analysis at 95 °C for 1 min. The melting curve was analyzed to confirm specific amplification and to ensure the absence of primer dimers. Data from four independent replicates, each performed in quadruplicate, were analyzed using the comparative threshold cycle (^ΔΔ^Ct) method. The GAPDH gene was used to normalize the data.

### 2.8. TAC and MDA Level Estimation in Spent IVM Medium

After 22 h of IVM, the spent medium from the control and treatment groups was collected and stored at −20 °C until analysis. The concentrations of TAC and MDA were measured at wavelengths of 505 and 534 nm, respectively, in three runs using commercial kits (Bio-diagnostic, Giza, Egypt) and spectrophotometry. All procedures were carried out according to the manufacturer’s instructions.

### 2.9. Statistical Analysis

The normality of the data distributions was evaluated by the Kolmogorov–Smirnov test. Oocyte recovery rate and differences between culturable and non-culturable oocytes are presented as mean ± SEM and were analyzed by an independent samples *t*-test. Cumulus cell expansion and nuclear maturation data are presented as percentages and analyzed by one-way ANOVA followed by Tukey’s post hoc test and a Chi-squared test, respectively; *p* < 0.05 was considered significant. The IBM SPSS 27.0 Software Package was used to conduct the statistical analyses (IBM Corp., New York, NY, USA). Gene expression data are presented as mean ± SEM and were analyzed by one-way ANOVA followed by Tukey’s multiple comparisons test; *p* < 0.05 was considered statistically significant. GraphPad Prism 5.0 was used for gene expression data analysis, as well as for the plotting.

## 3. Results

### 3.1. Oocyte Recovery Rate and Grading

In the present study, a total of 634 oocytes were recovered from 346 ovaries with a recovery rate of 2.02 ± 0.23 oocyte/ovary. Out of those collected oocytes, 426 (65.92%) were selected for IVM (culturable) and 208 (34.08%, *p* < 0.001) were discarded (non-culturable, Table 2).

### 3.2. The Effect of EMD-300^®^ and EMP3-H200^®^ Supplementation During IVM of Buffalo COCs on Cumulus Cell Expansion

**Figure 1:**
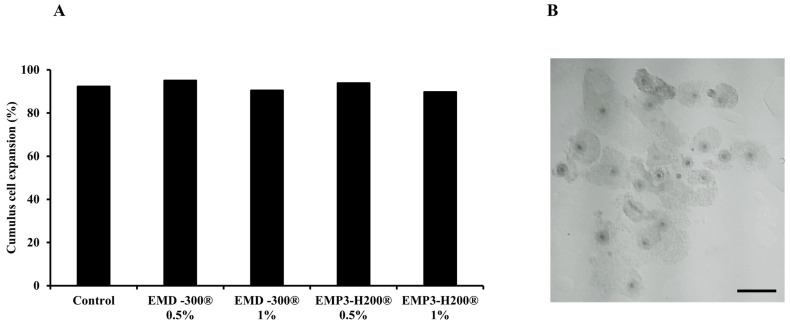
Cumulus cell expansion following in vitro maturation of buffalo oocytes matured in IVM medium supplemented with 0.5% or 1.0% of EMD-300^®^ or EMP3-H200^®^. The control group represents those oocytes that were subjected to IVM without EMD-300^®^ or EMP3-H200^®^ supplementation. Data are presented as percentages (n = 21–130 COCs/group) (**A**). The picture shows buffalo COCs following IVM with expanded cumulus cells (scale bare = 100 µM) (**B**).

No significant differences were observed in the percentage of oocytes with expanded cumulus cells (Figure 1B) between treated (0.5% or 1.0% of EMD-300^®^ or EMP3-H200^®^) and control groups (Figure 1A).

### 3.3. The Effect of EMD-300^®^ and EMP3-H200^®^ Supplementation During IVM of Buffalo Oocytes on Nuclear Maturation Rates

**Figure 2:**
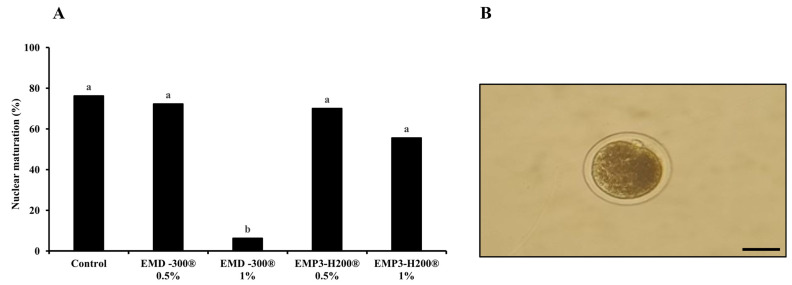
Nuclear maturation of buffalo oocytes following in vitro maturation of COCs in IVM medium supplemented with 0.5% or 1.0% of EMD-300^®^ or EMP3-H200^®^. The control group represents those oocytes that were subjected to IVM without EMD-300^®^ or EMP3-H200^®^ supplementation. Data are presented as percentages. ^a,b^ denote a significant difference at *p* < 0.001 (n = 9–21 oocytes/group) (**A**). The picture shows a buffalo oocyte following IVM with an extruded 1st polar body (scale bare = 100 µM) (**B**).

As shown in Figure 2A, supplementation of IVM medium with 1.0% EMD-300^®^ significantly (*p* < 0.001) reduced the nuclear maturation rates (oocytes with extruded 1st Pb; Figure 2B) compared to the other groups (6.25% for 1.0% EMD-300^®^, 55.55% for 1.0% EMP3-H200^®^, 70.00% for 0.5% EMP3-H200^®^, 72.22% for 0.5% EMD-300^®^, and 76.19% for the control). Furthermore, nuclear maturation rate was lower in the 1.0% EMP3-H200^®^ group compared to those treated with 0.5% of both compounds and the control group; however, the difference was not significant (Figure 2A). Therefore, 0.5% of EMD-300^®^ and EMP3-H200^®^ were used in gene expression analysis and the determination of TAC and MDA levels experiments.

### 3.4. The Effect of EMD-300^®^ and EMP3-H200^®^ Supplementation During IVM of Buffalo COCs on the Expression Pattern of Oxidative Stress Response-Associated Genes

**Figure 3:**
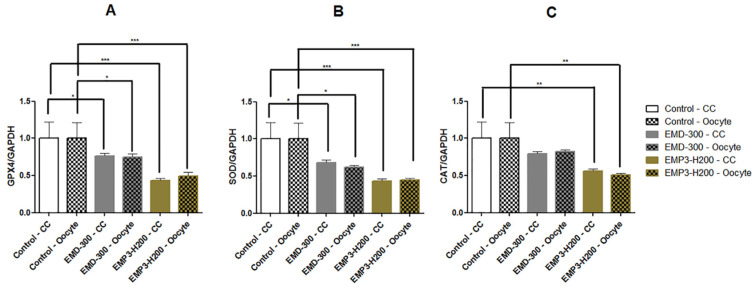
Expression patterns of the GPX4 (**A**), SOD (**B**), and CAT (**C**) genes in buffalo oocytes and cumulus cells (CC) following in vitro maturation in IVM medium supplemented with 0.5% EMD-300^®^ or 0.5% EMP3-H200^®^ compared to the control (oocytes that were subjected to IVM without EMD-300^®^ or EMP3-H200^®^ supplementation). Data are presented as mean ± SEM. * *p* < 0.05, ** *p* < 0.01, *** *p* < 0.001 (n = 4).

A significant decrease in the expression of the GPX4 and SOD genes was noted in oocytes and cumulus cells in the EMD-300^®^ and EMP3-H200^®^ supplemented groups (*p* < 0.05 and *p* < 0.001, respectively) compared to the control group (Figure 3A and Figure 3B, respectively). In addition, a significant decrease (*p* < 0.01) in CAT gene expression was observed in the oocytes and cumulus cells of EMP3-H200^®^ treated group compared to the control ones. On the other hand, there was no significant difference in the expression levels of the CAT gene between the EMD-300^®^ treated and control groups (Figure 3C).

### 3.5. The Effect of EMD-300^®^ and EMP3-H200^®^ Supplementation During IVM of Buffalo COCs on the Expression Pattern of Apoptotic and Endoplasmic Reticulum Stress-Associated Genes

**Figure 4:**
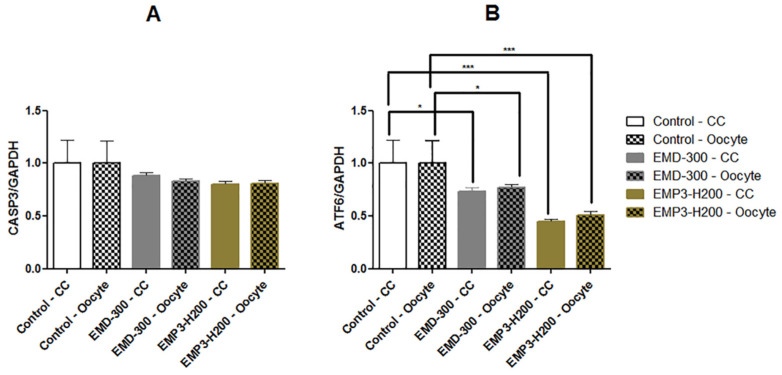
Expression patterns of the CASP3 (**A**) and ATF6 (**B**) genes in buffalo oocytes and cumulus cells (CC) following in vitro maturation in IVM medium supplemented with 0.5%EMD-300^®^ or 0.5% EMP3-H200^®^ compared to the control (oocytes that were subjected to IVM without EMD-300^®^ or EMP3-H200^®^ supplementation). Data are presented as mean ± SEM. * p < 0.05, *** p < 0.001 (n = 4).

No significant differences were observed in the expression levels of the CASP3 gene between the treated and control groups in both oocytes and cumulus cells (Figure 4A). However, the expression level of the ATF6 gene was significantly lower in the EMD-300^®^ (*p* < 0.05) and EMP3-H200^®^ (*p* < 0.001) supplemented groups than in the control group for both oocytes and cumulus cells (Figure 4B).

### 3.6. The Effect of EMD-300^®^ and EMP3-H200^®^ Supplementation During IVM of Buffalo COCs on the Expression Pattern of the OCT4 Gene

**Figure 5:**
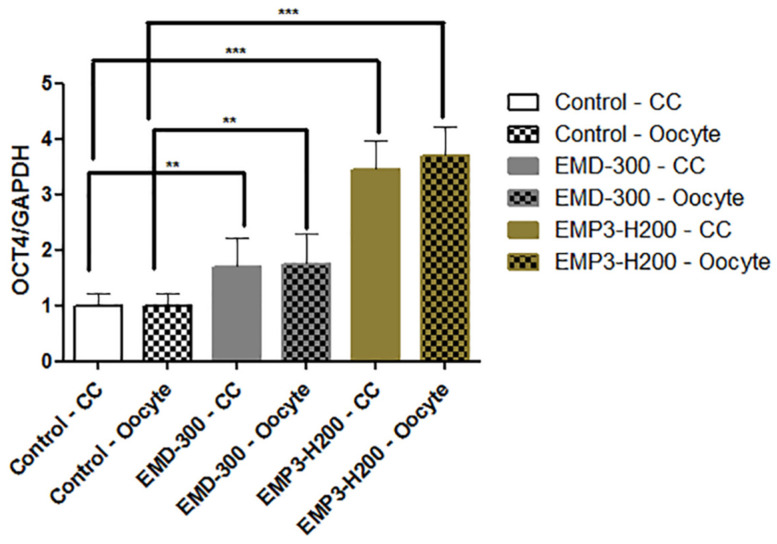
Expression pattern of the OCT4 gene in buffalo oocytes and cumulus cells (CC) following in vitro maturation in IVM medium supplemented with 0.5% EMD-300^®^ or 0.5% EMP3-H200^®^ compared to the control (oocytes that were subjected to IVM without EMD-300^®^ or EMP3-H200^®^ supplementation). Data are presented as mean ± SEM. ** p < 0.01, *** p < 0.001 (n = 4).

As shown in Figure 5, a significant increase in the expression level of the OCT4 gene was observed in both oocytes and cumulus cells for the EMD-300^®^ and EMP3-H200^®^ supplemented groups compared to the control one (*p* < 0.01 and *p* < 0.001, respectively).

### 3.7. Levels of TAC and MDA in Spent IVM Medium

The TAC level was higher in the 0.5% EMD-300 group than in 0.5% EMP3-H200^®^ and control groups. However, MDA was lower in 0.5% EMD-300 group than in other groups. The differences between the groups were not significant (Table 3).

## 4. Discussion

Oocyte quality and IVM are crucial for successful fertilization and in vitro embryo production. However, during in vitro culture, oocytes are exposed to high levels of ROS, which induces oxidative stress and subsequently impairs developmental competence. Oxidative stress during IVM occurs mainly due to the inability of oocytes to maintain the balance between the antioxidant defense mechanism and the excessive production of ROS. In conventional IVM, the oocytes are cultured under higher oxygen tension levels (3 to 4 times) than that present in vivo inside the ovarian follicles of the female reproductive tracts [43]. This can explain the notion of oocyte failure in mitigating the oxidative stress that occurs during IVM causing damage for oocytes and a decrease in their developmental competence after IVF [44]. In addition, the high lipid contents in buffalo oocytes render them highly susceptible to oxidative stress [5,45]. Hence, inclusion of antioxidants during IVM is imperative to enhance oocyte developmental competence in this species. Herein, we found that supplementation of IVM medium with novel naturally flavonoid-enriched antioxidant nanoformulations, namely, EMD-300 and EMP3-H200^®^, enhanced nuclear maturation rate, antioxidant capacity, and the expression levels of genes associated with oxidative stress and the development potential of buffalo oocytes.

It is well known that the oocyte recovery rate is low in buffalo compared to cattle mainly due to the low number of primordial follicles present in the buffalo ovary (15,000 to 19,000) [46]. In the present study, the oocyte recovery rate was higher than that reported by Yousaf and Chohan (2003) [47], (2.02 vs. 1.82 oocyte/ovary). However, it was comparable to those reported by Shahid et al., (2014) [48] (2.65 oocyte/ovary).

Nanoparticles possess distinct characteristics, including high chemical bioactivity and reactivity and the ability to penetrate cells, tissues, and organs and to enhance bioavailability [31]. It is well known that the size of the NPs significantly influences their ability to penetrate various membranes [31]. For instance, smaller NPs exhibit greater penetration and achieve higher cellular uptake than larger ones [31]. Analysis of oocyte maturation can give an indication of the perturbations that occur to the oocytes during in vitro culture. Cumulus expansion is necessary for meiotic maturation and acquiring developmental competence. Cumulus expansion is based on the extracellular matrix that synthesized by the cumulus cells. Hyaluronic acid is the most abundant component of this extracellular matrix [7,8,9]. Herein, we did not find significant differences in cumulus cell expansion between the EMP3-H200^®^ and EMD-300^®^ supplemented groups and the control one. Moreover, we found comparable nuclear maturation rates between the 0.5% EMP3-H200^®^ and 0.5% EMD-300^®^ supplemented groups (70.00% and 72.22%, respectively) despite the differences in their particle size (173 nm and 490 nm, respectively). This can be explained on the basis of irrespective to the size of a NP, its penetration ability may differ according to cell types and the properties of their membranes. To prove this hypothesis, further experiments are warranted, which require many oocytes, and this is one of the limitations in the current study with buffalo IVEP. We also noticed a substantial reduction in nuclear maturation rates in the 1.0% EMD-300^®^ and 1.0% EMP3-H200^®^ supplemented groups compared to other ones (Figure 2A). This can be explained by fact that these compounds had low efficacy at 1.0%, and their impacts on oocyte nuclear maturation were impeded at the higher concentration [36,49]. In line with our study, the effects of flavonoid-enriched antioxidants, such as kaempferol, quercetin, resveratrol, and nobiletin, on oocyte nuclear maturation and in vitro embryo development have been documented in different species, including buffalo [26], cattle [27,28], and sheep [29].

Under normal conditions, cells sustain their ROS levels in equilibrium [19], while during IVM, the oocytes may experience changes in redox equilibrium, which have adverse impacts on oocyte maturation and developmental competence [20,21]. However, studies have demonstrated that the addition of antioxidants into the culture medium reduces the harmful effects of ROS during IVM and subsequent protects the oocytes and in vitro produced embryos against oxidative injury [21,26]. In the oocyte, the major ROS scavenger system is GSH, which uses reducing power provided by oxidative metabolism [19]. Furthermore, superoxide dismutase helps in eliminating superoxide radicals, whereas hydrogen peroxide (H_2_O_2_), the byproduct of SOD activity, is processed by CAT and GPX, leading to a decrease in lipid hydroperoxides [50]. Herein, we found that supplementation of IVM medium with 0.5% EMD-300^®^ and 0.5% EMP3-H200^®^ reduced the expression levels of the GPX4, SOD, and CAT genes in both oocytes and cumulus cells, which is in agreement with the use of other flavonoids such as resveratrol [51], quercetin, taxifolin [52], and nobiletin [28].The reduction in the expression levels of the GPX4, SOD, and CAT genes in the EMD-300^®^ and EMP3-H200^®^ groups assure the antioxidant effects of these compounds and their role in neutralizing ROS and protecting the oocytes against oxidative damage, such that a reduction in ROS formation requires less endogenous antioxidants to neutralize the free radicles. Nonetheless, further studies are necessary to understand the antioxidant mechanisms of EMD-300^®^ and EMP3-H200^®^ and their effects on oocyte developmental potential. Opposed to our findings, previous studies reported an increase in the expression levels of endogenous antioxidant enzymes in relation to utilization of different antioxidants in the oocyte culture medium [42,53,54,55,56,57,58,59,60]. Interestingly, a previous study reported that a reduction in SOD2 mRNA transcription in the oocyte is associated with improvement in oocyte developmental competence [6]. Notably, we found that both 0.5% EMD-300^®^ and 0.5% EMP3-H200^®^ inclusion during IVM enhanced the levels of TAC and decreased the levels of MDA in spent IVM medium in comparison to the control (Table 3). These results assure that EMD-300^®^ and EMP3-H200^®^ at 0.5% could have an antioxidant effect and the ability to protect the oocytes against oxidative stress during IVM. In agreement with our results, previous studies showed that supplementation of IVM with other antioxidants, including melatonin, zinc chloride, and sodium selenite, is associated with an increase in TAC and a decrease in MDA levels in dromedary camels [61] and buffalo [62].

It is well known that the apoptotic process affects the viability and survival rate of oocytes, consequently impacting embryo development [63]. CASP3 is a crucial enzyme in the last stages of cell apoptosis, and its activated form serves as the main mediator for the apoptotic process [64]. Therefore, a lower the expression of the CASP3 gene indicates better oocyte quality. Herein, we found that the levels of CASP3 gene expression were lower in the treated groups than in control (Figure 4A). These observations indicate that both EMD-300^®^ and EMP3-H200^®^ may have antiapoptotic impacts and could protect the oocytes against apoptotic changes. The effect of antioxidant supplementation during IVM on the levels of CASP3 gene expression is still controversial and depends on the species. For instance, it has been shown that inclusion of melatonin, a potent antioxidant, during IVM of buffalo oocytes significantly reduced the levels of CASP3 gene expression in matured oocytes [65]. However, in pigs, Xiang et al. reported that adding astaxanthin as an efficient antioxidant did not significantly affect CASP3 activity in vitrified IVM oocytes [66]. ATF6 is an important sensor in the unfolded protein response (UPR) pathway. This gene is triggered in response to endoplasmic reticulum (ER) stress [67]. Our results revealed a significant reduction in the expression of the ATF6 gene in both oocytes and cumulus cells treated with EMD-300^®^ or EMP3-H200^®^ (Figure 4B). In response to stress, up regulation of ATF6 gene expression enhances the ER’s ability to manage cellular stress. ATF6 plays an essential role in mitigating protein misfolding and ensuring the proper function of the ER [68]. Under mild to moderate stress conditions, the activation of ATF6 in conjunction with other UPR pathways facilitates cell survival by enhancing protein quality control. However, under prolonged or severe stress, ATF6 can initiate pro-apoptotic signaling, leading to the elimination of irreparably damaged cells [69]. Thus, an increase in ATF6 gene expression in treated groups indicates the higher level of stress that oocytes experience during IVM. The influence of antioxidants such as resveratrol on the levels of ATF6 gene expression in in vitro produced embryos has been previously documented [42]. Previous studies showed that suppression of UPR signaling with ER stress inhibitors improved oocyte maturation and early embryonic development in buffalo and cattle [70,71].

Moreover, we observed over expression in the levels of OCT4 gene in both oocytes and cumulus cells treated with 0.5% EMD-300^®^ or 0.5% EMP3-H200^®^. OCT4 has been identified as one of the main regulators for pluripotency during embryonic development [72]; it has antiapoptotic effect [73]. Extensive research demonstrated that OCT4 plays a crucial role in the acquisition of oocyte developmental competence [74,75]. For example, previous studies noticed that low levels of OCT4 in bovine oocytes are associated with lower developmental potential [76]. In accordance with our findings, the levels of OCT4 gene expression were positively correlated with utilizing various antioxidants in IVM media of buffalo [33,77] and cattle [78,79,80]. The effect of EMD-300^®^ and EMP3-H200^®^ on fertilization, cleavage, blastocyst rates, implantation rates, livebirth ratio following IVF, in vitro embryo culture, and embryo transfer were not evaluated in the current study, as our main focus was to investigate if these compounds, as novel flavonoid-enriched antioxidant nanoformulations, could be utilized during IVM to improve oocyte quality and maturation rates in buffalo, as oocyte IVM is the corner stone for IVEP. Future studies are warranted to test the effects of these compounds on IVF and embryo development rates.

## 5. Conclusions

Our findings suggest that supplementing IVM medium with EMD-300^®^ and EMP3-H200^®^, flavonoid-enriched antioxidant nanoformulations, at 0.5% improved nuclear maturation of buffalo oocytes as compared to 1.0%. Additionally, we reported that these compounds have antioxidant properties, as demonstrated by changes in the expression levels of genes associated with oxidative stress (SOD2, GPX4, CAT), apoptosis (CASP3), and endoplasmic reticulum stress (ATF6). Furthermore, both EMD-300^®^ and EMP3-H200^®^ at 0.5% increased the expression levels of OCT4. Our results highlight the potential impacts of flavonoid-enriched antioxidant nanoparticle supplementation on oocyte quality and in vitro culture in buffalo.

## Figures and Tables

**Table 1:** List of primers chosen for qRT-PCR analysis.

Gene	Sequence 5′–3′	Accession No.	Product Size (bps)	Annealing °C
GPX4	F: GCTCATTGAGAACGTAGCAT R: GTACTTCAGGCAATTCAGGAT	NM_174076.3	175	55
SOD	F: GATACAGTCGTGGTAACTGGAT R: TCTCCTGAGAGTGAAATCAGA	NM_174615.2	248	54
CAT	F: TCTCCACTGTTGCTGGAGAAT R: TGCGTTTGAGGGTTTCTCTT	NM_001035386.2	187	54
ATF6	F: AAGACAAGCCCATCATTGGT R: TGATTGTTTTTGCTGGAACG	XM_024989877.1	162	51
CASP3	F: GACTGTGGTATTGAGACAGACA R: CGTACTTTTTCAGCATCTCAC	XM_006075118.1	175	50
OCT4	F: CAGAAGAGGATCACACTAGGAT R: GTCTCTGCCTTGCATATCTC	NM_174580.3	212	53
GAPDH	F: CTACATGGTCTACATGTTCCAG R: CCTTCTCCATGGTAGTGAAGA	XM_006065800.1	200	50

CAT: catalase, GPX4: glutathione peroxidase 4, SOD: superoxide dismutase, ATF6: activating transcription factor 6, CASP3: caspase 3, OCT4: octamer-binding transcription factor 4, GAPDH: glyceraldehyde-3-phosphate dehydrogenase, and bps: base pairs.

**Table 2:** Recovery rate of the oocytes from buffalo ovaries.

Ovaries (n)	Oocytes (n)	Recovery Rate (Mean ± SEM)	Culturable Oocytes n (Mean% ± SEM)	Non-Culturable Oocytes n (Mean% ± SEM)
346	634	2.02 ± 0.23	426 (65.92 ± 2.61) ^a^	208 (34.08 ± 2.61) ^b^

Oocyte recovery rate was calculated as the mean of the number of recovered oocyte/ovary in nine replicates. Values with different superscripts within the same row denote significant differences at *p* < 0.001.

**Table 3:** The effect of EMD-300^®^ and EMP3-H200^®^ supplementation during IVM of buffalo COCs on total antioxidant capacity (TAC) and malondialdehyde (MDA) levels in spent medium.

Treatment	TAC (mmol/L)	MDA (nmol/mL)
Control	0.29 ± 0.17	17.07 ± 3.91
EMD-300^®^ 0.5%	0.41 ± 0.19	8.46 ± 2.14
EMP3-H200^®^ 0.5%	0.35 ± 0.15	11.91 ± 4.74

Data are presented as mean ± SEM. No significant differences were observed between the groups (n = 3).

## Data Availability

All generated data are provided in this submission.
